# Fault Location of Generator Stator with Single-Phase High-Resistance Grounding Fault Based on Signal Injection

**DOI:** 10.3390/s25196132

**Published:** 2025-10-03

**Authors:** Binghui Lei, Yifei Wang, Zongzhen Yang, Lijiang Ma, Xinzhi Yang, Yanxun Guo, Shuai Xu, Zhiping Cheng

**Affiliations:** 1 Baihetan Hydropower Plant, Liangshan 615400, China; lei_binghui@ctg.com.cn (B.L.); yang_zongzhen@ctg.com.cn (Z.Y.); Ma_lijiang@ctg.com.cn (L.M.); 2 School of Electrical and Information Engineering, Zhengzhou University, Zhengzhou 450001, China; guoyanxun@zzu.edu.cn (Y.G.); xszzu2020@zzu.edu.cn (S.X.); zpcheng@zzu.edu.cn (Z.C.); 3 China Three Gorges International Corporation, Beijing 100006, China; yang_xinzhi@ctg.com.cn

**Keywords:** stator ground fault, grounding transition resistance, wavelet transform, singular value decomposition denoising

## Abstract

This paper proposes a novel method for locating single-phase grounding faults in generator stator windings with high resistance, which are typically challenging to locate due to weak fault characteristics. The method utilizes an active voltage injection technique combined with traveling wave reflection analysis, singular value decomposition (SVD) denoising, and discrete wavelet transform (DWT). A DC voltage signal is then injected into the stator winding, and the voltage and current signals at both terminals are collected. These signals undergo denoising using SVD, followed by DWT, to identify the arrival time of the traveling waves. Fault location is determined based on the reflection and refraction of these waves within the winding. Simulation results demonstrate that this method achieves high accuracy in fault location, even with fault resistances up to 5000 Ω. The method offers a reliable and effective solution for locating high-resistance faults in generator stator windings without requiring winding parameters, demonstrating strong potential for practical applications.

## 1. Introduction

Large generators are integral to the reliability and efficiency of hydroelectric power systems, and the shutdown of large generators caused by faults can significantly affect the normal operation of the power system. Stator winding ground faults in generators occur when the insulation layer is damaged, causing contact between the iron core and the windings [1,2]. Upon the occurrence of such faults, it is crucial that protection devices act immediately to prevent more extensive damage [3,4,5]. After isolating the fault, it is essential to employ reliable methods to accurately identify the fault location and perform repairs, thereby minimizing the losses associated with generator downtime [6,7].

The identification of fault locations in generator ground faults is typically closely related to the magnitude of the fault resistance. When the fault resistance is low, the fault characteristics are more apparent, making fault location relatively simple and precise. However, when the fault resistance is high, the fault characteristics become less distinct, which poses significant challenges in fault location. Specifically, when the stator winding insulation layer is damaged, the contact between the stator windings and iron cores via a medium that behaves similarly to a semiconductor material may lead to generator ground faults with high grounding transition resistance.

Once protections are applied [8], there are numerous fault location techniques that can be utilized in both online and offline modes. Paper [9] proposes a fault location algorithm without transition resistance by analyzing conductor potentials in an online state. Paper [10] also proposes a universal algorithm for online stator winding ground fault location under working conditions. The online method proposed in [11] analyzes third harmonic voltage information to construct fault localization equations based on the equivalent circuit of third harmonic voltage. The optimal objective function is determined to locate the fault position. Although the method shows accuracy, the highest transition resistance that simulation tested is only 1 kΩ. All these methods rely heavily on precise generator parameters, and without accurate data, accurate fault location cannot be achieved [12].

In practice, when a generator fails, it must be shut down for maintenance. The paper [13] develops experiment platform for the online and offline location methods of generator stator core. However, the stator core fault is different from the stator winding fault. Regarding offline fault location methods for stator windings, Paper [14] proposes three different methods and shows experiment results. However, its tolerance ability of fault resistance is only 1.25 kΩ.

The traveling wave method has been widely applied in fault location for transmission lines. However, the fault resistance in transmission lines typically remains below 500 Ω [15], whereas the fault resistance in generator single-phase ground faults can exceed 1000 Ω [16]. Moreover, existing generator fault location methods face challenges such as difficulty in determining accurate generator parameters, difficulty in recognizing fault characteristics when the fault resistance is high, complexity in operation, and the potential for further damage to the generator.

To address these issues, this paper proposes a fault location method, which is based on the traveling wave process in the windings. This method does not require the determination of generator parameters and can accurately locate the fault position even at a fault resistance of 5000 Ω. Furthermore, it is simple to operate and does not cause secondary damage to the generator.

This paper is composed of the following sections: Section 2 introduces the reflection and refraction rules of the injected signal in the stator windings. It analyzes the variation rules of the voltage and current signals collected at both terminals of the stator windings. Section 3 introduces an auxiliary location method based on features extracted from the reconstructed signals. Section 4 verifies the location method and auxiliary method for locating extremely high impedance faults. In the end, Section 5 concludes the proposed method.

## 2. Analysis and Identification of Traveling Wave

Following a generator shutdown due to a fault, the faulty phase and branch can be identified through protection equipment, which is not discussed in this paper. Instead, this paper aims to locate the fault point within the fault branch.

### 2.1. Traveling Wave Propagation Process in Fault Branch

**Figure 1:**
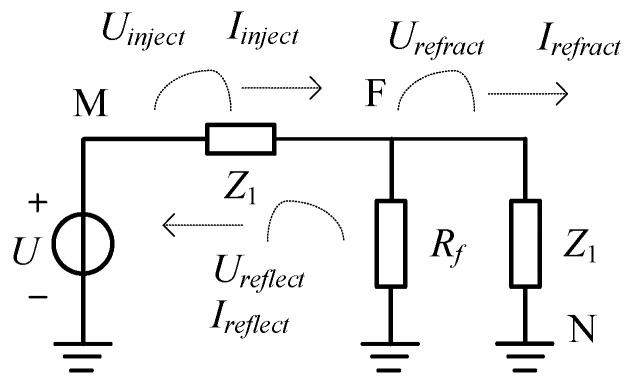
Boundary circuit of the stator winding.

Upon injecting a voltage signal into the winding, the traveling wave propagates within the winding. Due to the impedance discontinuity at the fault point, refraction and reflection occur at the fault location, with the waves propagating towards both the non-injection terminal and the injection terminal. The schematic diagram of the injected voltage traveling wave and its reflection/refraction is shown in Figure 1. Here, *U_inject_* and *I_inject_* represent the injected voltage and current traveling waves, respectively; *U_reflect_* and *I_reflect_* represent the reflected voltage and current traveling waves at the fault point, respectively; and *U_refract_* and *I_refract_* represent the refracted voltage and current traveling waves at the fault point, respectively. The M and N correspond to the two terminals of the stator winding, while point F represents the fault location. The traveling wave signal is injected by applying a DC voltage with an amplitude of *U*.

The impedance of the stator winding shown in Figure 1 is *Z*_1_. The equivalent impedance of the conductor after the fault point, *Z*_2_, which is the parallel connection of the fault resistance *R_f_* and stator winding impedance *Z*_1_ is

(1) $$Z_{2}=Z_{1}//R_{f}=\frac{Z_{1}R_{f}}{Z_{1}+R_{f}}$$

Considering the impedance of stator winding *Z*_1_ and fault resistance *R_f_*_,_ representing the voltage and current at the fault point with *U_f_* and *I_f_* [17], then

(2) $$\left\{\begin{array}{l} U_{f}=U_{inject}+U_{reflect}=U_{refract}\\ I_{f}=I_{inject}-I_{reflect}=\frac{U_{inject}}{Z_{1}}-\frac{U_{reflect}}{Z_{1}}=\frac{U_{refract}}{Z_{2}} \end{array}\right.$$

The formulations for the corresponding refracted and reflected traveling waves can be obtained in Equations (3) and (4).

(3) $$\left\{\begin{array}{l} U_{refract}=U_{inject}\frac{2Z_{2}}{Z_{1}+Z_{2}}\\ U_{reflect}=U_{inject}\frac{Z_{2}-Z_{1}}{Z_{1}+Z_{2}} \end{array}\right.$$

(4) $$\left\{\begin{array}{l} I_{refract}=I_{inject}\frac{2Z_{2}}{Z_{1}+Z_{2}}\\ I_{reflect}=I_{inject}\frac{Z_{2}-Z_{1}}{Z_{1}+Z_{2}} \end{array}\right.$$

Since *Z*_2_ < *Z*_1_, the refraction coefficient at the fault point is positive, causing the refracted traveling wave to retain the same polarity as the injected traveling wave. Conversely, because the reflection coefficient is negative, the reflected traveling wave has the opposite polarity to that of the injected traveling wave.

**Figure 2:**
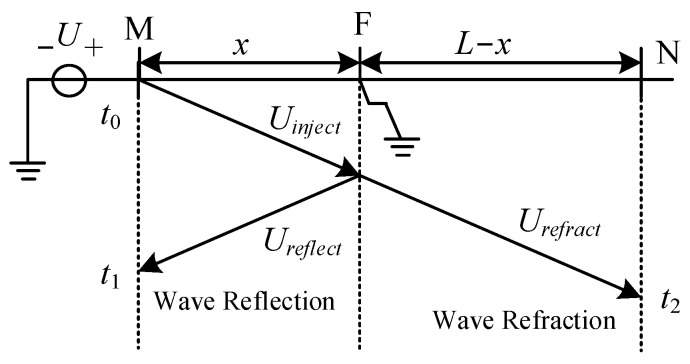
Traveling wave propagation of injected signal.

Figure 2 shows the propagation process of the injected signal within the stator winding. The distance from the fault point F to the terminal M is called the fault distance *x*, and the total length of the stator winding is *L*. A DC voltage of *U* is injected into the terminal M at time *t*_0_. The traveling wave propagates from terminal M towards terminal N, encountering refraction and reflection at the fault point F. The reflected wave returns to terminal M and arrives at time *t*_1_, causing signal mutation at terminal M. The refracted wave *U_refract_* continues to propagate through the fault point, and it reaches terminal N at time *t*_2_, causing signal mutation of terminal N. The current mutation of terminal M at *t*_1_, marked as *ΔI_M_*, and the voltage mutation of terminal N at *t*_2_, marked as *ΔU_N_*, are given as follows [18].

(5) $$\left\{\begin{array}{l} \Delta I_{M}=H\frac{Z_{2}-Z_{1}}{Z_{1}+Z_{2}}I_{\mathrm{inject}}\\ \Delta U_{N}=H\frac{2Z_{2}}{Z_{1}+Z_{2}}U_{inject}\\ H=\frac{1-k_{a}x}{1+\tau_{a}xs}e^{-\frac{x}{v}s} \end{array}\right.$$

where *H* is the propagation function, and *k_a_* and *τ_a_* are the attenuation coefficient and dispersion coefficient, respectively.

**Figure 3:**
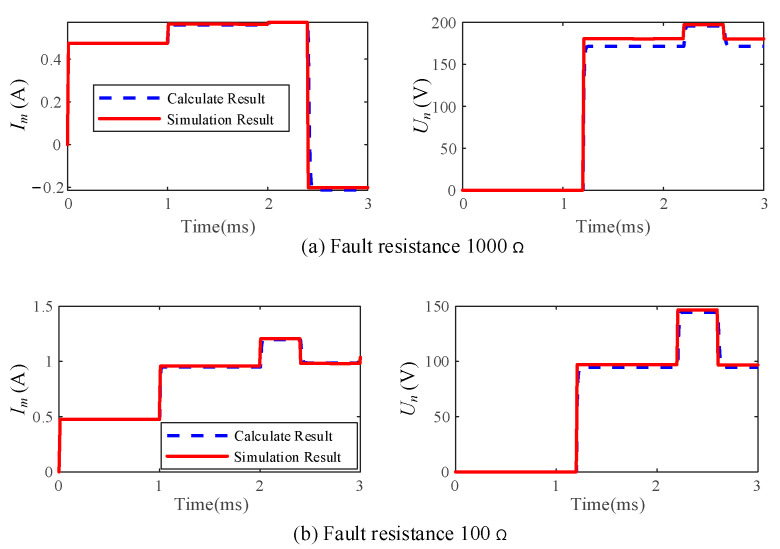
Schematic diagram of waveform of *I_m_* and *U_n_*. (**a**) Fault resistance 1000 Ω. (**b**) Fault resistance 100 Ω.

Taking a winding with a length of 360 m as an example, the fault distance and the injection DC voltage were set to 150 m and 100 V, respectively. The fault resistances are 100 Ω and 1000Ω, respectively, and the calculation and simulation results are given in Figure 3a,b.

As shown in Figure 3, the simulation and calculation curves almost coincided, validating the correctness of the former calculation methods. When the refracted wave arrives at terminal N, the voltage abruptly increases, and the voltage variation increases with fault resistance. When the reflected wave arrives at terminal M, the current abruptly increases, and the current variation decreases with fault resistance. Thus, the detection of voltage refracted waves under the small fault resistance and the detection of current reflected waves under large fault resistance should be taken carefully. In fact, the fault location of generator stator with small fault resistance can be easily achieved by existing methods, thus, this paper focuses on the fault location of generator stator with large fault resistance.

### 2.2. Discrete Wavelet Transform

After the injection, the signal processing is important for obtaining the signal characteristic. In [19], the principal component analysis is used to construct the comprehensive figure of merit for the transducer. In [20], the dynamic root mean square of the acoustic emission signal is revealed to serve for the fault diagnosis of high-speed train axle box bearing. In [21], an adaptive CYCBDβ (ACYCBDβ) was proposed, which is a new method for early bearing fault diagnosis. It can accurately extract the early fault signals, even under strong non-Gaussian noise interference, without requiring any prior information about the fault cycle, and adaptively determines the appropriate filter length. In this paper, the wavelet transform is used to extract the signal mutation and arrival time of traveling wave.

To better identify the arrival time of the reflected wave from the fault point, wavelet transform is used to analyze the traveling waves and extract the signal mutation caused by the wave arrival [22]. Wavelet transform represents the signal *f*(*t*) by stretching and translating a single function (usually also called the mother wave) *Ψ*(*t*). Wavelet transform has very good time–frequency domain characteristics and can be used to capture signal mutations. Taking *Ψ*(*t*) as the mother wave, the continuous wavelet transform of the signal *f*(*t*) is expressed as

(6) $$(W_{\psi }f)(a,b)=\frac{1}{\sqrt{a}}\int_{-\infty }^{+\infty }f(t)\psi^{*}(\frac{t-b}{a})dt$$

where *a* and *b*, respectively, correspond to the scale parameter and position parameter. The signal *f*(*t*) can also be reconstructed by its wavelet transform, with the reconstruction formula as follows:

(7) $$f(t)=\frac{1}{C_{\psi }}\int_{-\infty }^{+\infty }\int_{-\infty }^{+\infty }\left(W_{\psi }f\right)(a,b)\psi_{a,b}\frac{da}{a^{2}}db$$

In practice, signals collected are often discreet in nature. To address this issue, Mallat’s algorithm implements the discrete wavelet transform through a series of filtering and boundary conditions [23]. The continuous wavelet transform described in Equation (6) can be transformed into a discrete wavelet transform according to Mallat’s algorithm, as shown in Equation (8).

(8) $$(W_{\psi }f)(\frac{1}{2^{j}},\frac{k}{2^{i}})=\int_{-\infty }^{+\infty }f(t)2^{j}\psi^{*}(2^{j}t-k)dt$$

**Figure 4:**
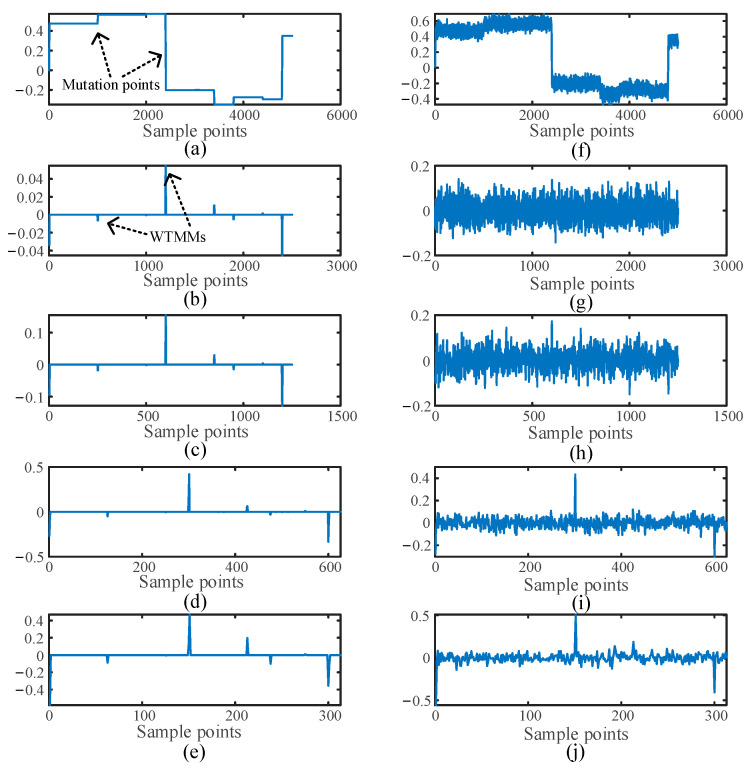
Discrete wavelet transforms. (**a**–**e**) Original signal and discrete wavelet transform (**f**–**j**) 20 dB signal and discrete wavelet transform.

In Figure 4a–e correspond to the noise-free original signal and the first-, second-, third-, and fourth-level wavelet coefficients, respectively. Figure 4f–j correspond to the noisy original signal and the first-, second-, third-, and fourth-level wavelet coefficients, respectively. Since the first-level wavelet coefficients contain the high-frequency components of the signal, which match the high-frequency traveling wave signal analyzed in this paper, the first-level wavelet coefficients are used to identify the signal mutation points and determine the arrival time of the traveling wave.

Figure 4a,b precisely demonstrate that wavelet transform can effectively identify the signal’s abrupt change points. The degree of signal mutation is directly related to the amplitude of the Wavelet Transform Modulus Maxima (*WTMM*). The discrete wavelet transform can precisely detect mutation points in the signal, where the *WTMM* correspond to signal mutation points [24].

The signals collected in engineering applications typically contain noise. By adding 20 dB of noise to the original signal shown in Figure 4f, and performing a wavelet transform, the resulting signal is illustrated in Figure 4g. The mutation points of noisy signals cannot be identified by WTMM. Therefore, appropriate denoising of the collected signals is essential.

### 2.3. Singular Value Decomposition Denoising

In practical applications, the traveling wave under high-resistance fault may be covered by noise, thus, denoising technology is important for the fault location. There are different denoising methods. For example, the paper [21] proposes adaptive maximum generalized Gaussian cyclostationarity blind deconvolution to exclude the interference of strong non-Gaussian noise.

This paper adopts the singular value decomposition (SVD) method to denoise the acquired voltage and current signals. A one-dimensional traveling wave signal can be represented as *E* = [*E*(1), *E*(2) … *E*(*t*)]. The collected discrete signal is transformed into a traveling wave information matrix, where *E*(1) to *E*(*q*) from the acquired signal are used as the first row of the matrix, *E*(2) to *E*(*q* + 1) are used as the second row of the matrix, and so forth, constructing a *p*-row *q*-column traveling wave information matrix *C*, as shown in Equation (9).

(9) $$C=\left[\begin{array}{cccc} E(1) & E(2) & \ldots & E(q)\\ E(2) & E(3) & \ldots & E(q+1)\\ \vdots & \vdots & & \vdots \\ E(p) & E(p+1) & \ldots & E(p+q-1) \end{array}\right]$$

where *p* ≥ 2, *q* ≥ 2, *p* and *q* are integers and *p + q* − 1 ≤ *t*.

Singular value decomposition of the traveling wave information matrix yields the singular value matrix and the corresponding eigenvector matrix. The components with larger singular values contain the traveling wave information, while the components with smaller singular values contain the noise information. By filtering out the smaller singular values and reconstructing the signal using the eigenvector matrix, the signal can be effectively denoised.

**Figure 5:**
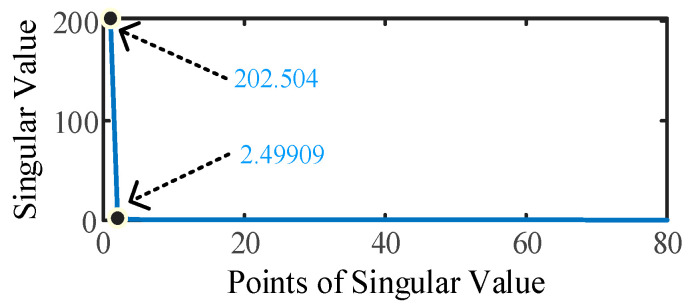
Singular value distribution.

Taking the traveling wave current from terminal M in Figure 3a as an example, 20 dB noise is added to the signal. The traveling wave information matrix *D* is constructed according to Equation (9). Matrix *D* is an 80 × 1900 matrix with 80 singular values. It can be observed from Figure 5 that when the 80 singular values are arranged in descending order, the second maximum singular value is 2.499, which is only 1.234% of the maximum singular value of 202.504.

We only retain the maximum singular value (MSV) and set all singular values smaller than the MSV to zero, as shown in Equation (10), where $\sigma_{i}$ represents the singular value. The denoised signal can then be reconstructed from the modified singular values.

(10) $$\sigma_{i}=\left\{\begin{array}{ll} \sigma_{i}, & \mathrm{if}\;\sigma_{i}=MSV\\ 0, & \mathrm{if}\;\sigma_{i}<MSV \end{array}\right.$$

**Figure 6:**
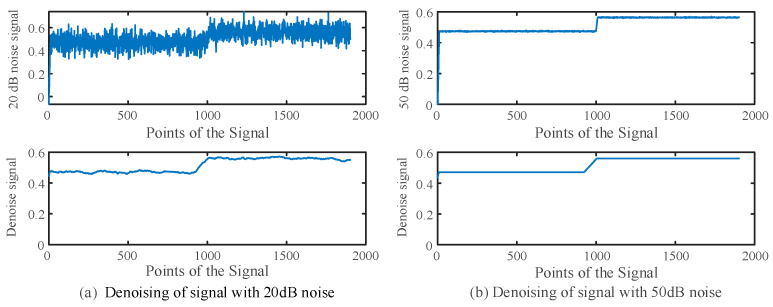
Denoising performances of different noises.

As shown in Figure 6, the denoising effect is demonstrated to be good when the noise is 20 dB or 50 dB. Nevertheless, even if the noise is not totally filtered out, the proposed location method can still achieve accurate fault location by multiple signal injections, which will be presented in Section 3.2.

## 3. Fault Location Scheme for Stator Windings

### 3.1. Fault Location Without High Fault Resistance

Since terminal M is directly connected to the voltage source, the voltage traveling wave is not obvious, but the current traveling wave change is significant. For terminal N, as it is open to ground, the current traveling wave change is not obvious, whereas the voltage traveling wave change is significant. Therefore, terminal M mainly collects current traveling wave data and terminal N mainly collects voltage traveling wave data.

The process of traveling wave refraction and reflection is shown in Figure 2. Due to the fault location necessarily being between terminals M and N, at time *t*_1_ the first reflected wave received at terminal M must have been reflected from the fault point. The first fault-refracted wave received at terminal N at time *t*_2_ must have been the traveling wave injected at terminal M and traveled through the complete stator winding. The fault distance, from terminal M to point F, is *x*. The total length of the winding is *L*, and the velocity of traveling wave is *v*. According to the propagation process of traveling waves, we can obtain Equation (11), where *t*_0_ is the instant of injecting signal.

(11) $$\left\{\begin{array}{l} t_{1}-t_{0}=\frac{2x}{v}\\ t_{2}-t_{0}=\frac{L}{v} \end{array}\right.$$

The injected traveling wave is composed of many components with different frequencies. Because of the frequency dependence characteristic, compared with the low-frequency components, the high-frequency components propagate faster and attenuate more severely, causing the dispersion effect of traveling waves. In addition, the velocity of traveling wave changes under different operation conditions of the stator windings, such as the material degradation and insulation damage. Therefore, it is difficult to obtain the actual value of traveling wave velocity. In this paper, the proposed method does not need to obtain the traveling wave velocity before the fault location. By solving Equation (11), the fault distance *x* is directly obtained as follows.

(12) $$x=\frac{L}{2}\frac{t_{1}-t_{0}}{t_{2}-t_{0}}$$

Considering that stator winding faults are usually associated with fault resistance, the amplitude of the reflected wave is directly related to the fault resistance and requires careful attention. In contrast, the refracted wave induces a noticeable transition from non-existence to presence at the non-injection terminal, making it relatively prominent. Therefore, the key to this method lies in accurately identifying the moment when the first abrupt change occurs in the signal at the injection terminal.

### 3.2. Fault Location with High Fault Resistance

When a single-phase low-resistance grounding fault occurs in the generator stator winding, the reflected and incident waves are evident. However, when a single-phase high-resistance grounding fault occurs, with *R_f_* ≈ +∞ corresponding to *Z*_2_ ≈ *Z*_1_, the reflected and incident waves are, respectively, given by

(13) $$\left\{\begin{array}{l} U_{refract}=U_{inject}\frac{2Z_{2}}{Z_{1}+Z_{2}}\approx U_{inject}\\ U_{reflect}=U_{inject}\frac{Z_{2}-Z_{1}}{Z_{1}+Z_{2}}\approx 0 \end{array}\right.$$

(14) $$\left\{\begin{array}{l} I_{refract}=I_{inject}\frac{2Z_{2}}{Z_{1}+Z_{2}}\approx I_{inject}\\ I_{reflect}=I_{inject}\frac{Z_{2}-Z_{1}}{Z_{1}+Z_{2}}\approx 0 \end{array}\right.$$

By comparing Figure 3 and Figure 5, it is evident that even with an appropriate threshold set for signal denoising, slight fluctuations in what was originally a stable signal are inevitable [25]. These minor fluctuations, after discrete wavelet transformation, evolve into numerous small *WTMM* points. According to Equations (13) and (14), the reflected wave becomes rather weak when the fault resistance is excessively large. After denoising, the small fluctuations caused by the denoise process and the variation caused by the reflected wave are mixed, which hinders the fault location. To solve this problem, after performing the first injection of terminal M, the voltage signal is injected into terminal N of the stator winding.

According to Figure 2, at time *T*_0_, a signal is re-injected from terminal N, with the reflected wave received at terminal N at time *T*_1_ and the refracted wave at terminal M at time *T*_2_, as expressed by Equation (15).

(15) $$\left\{\begin{array}{l} t_{2}-t_{0}=T_{2}-T_{0}\\ t_{1}-t_{0}+T_{1}-T_{0}=2(t_{2}-t_{0}) \end{array}\right.$$

Assuming that the multiple reflected wave arrival times *t*_11_, *t*_12_, *t*_13_… have been determined when the signal is injected from terminal M, then injecting a signal from terminal N will also yield multiple traveling wave arrival times, denoted as *T*_11_, *T*_12_, *T*_13_ and so on. Since the time axes corresponding to both injections are different, a conversion is necessary using Equation (16), so that the multiple arrival times *T*_11_, *T*_12_, *T*_13_… obtained from the signal injection at terminal N correspond to the recalculated times *t*_11_′, *t*_12_′, *t*_13_′…; *j* represents the number of suspected points of arrival of the reflected wave obtained by analyzing after injecting signals into terminal N.

(16) $$t_{1j}^{\prime }=t_{0}-T_{1j}+2T_{2}-T_{0}$$

Since the fault location is fixed, the arrival time of the traveling wave should also be a determined value. The position of one of the multiple fault points ×1, ×2, ×3… corresponding to the multiple arrival times obtained from injecting the signal at terminal M will inevitably be close to one of the fault points ×1′, ×2′, ×3′… calculated from the signal injection at terminal N. The average of the two corresponding positions will be the actual fault location, with the rest being false fault locations caused by denoised fluctuations.

### 3.3. Fault Location Procedure

The proposed method works normally with different values of injected DC voltage, as long as the voltage and current sensors have sufficient accuracy and the generator is able to endure the voltage and current stresses. In this paper, the large-scale generator is considered. The injected voltage of 100 V is selected and thus the injected current is no more than 1 A, and the voltage and current last only a few milliseconds, which satisfies the safe constraint and is non-damaging for the large-scale generator.

**Figure 7:**
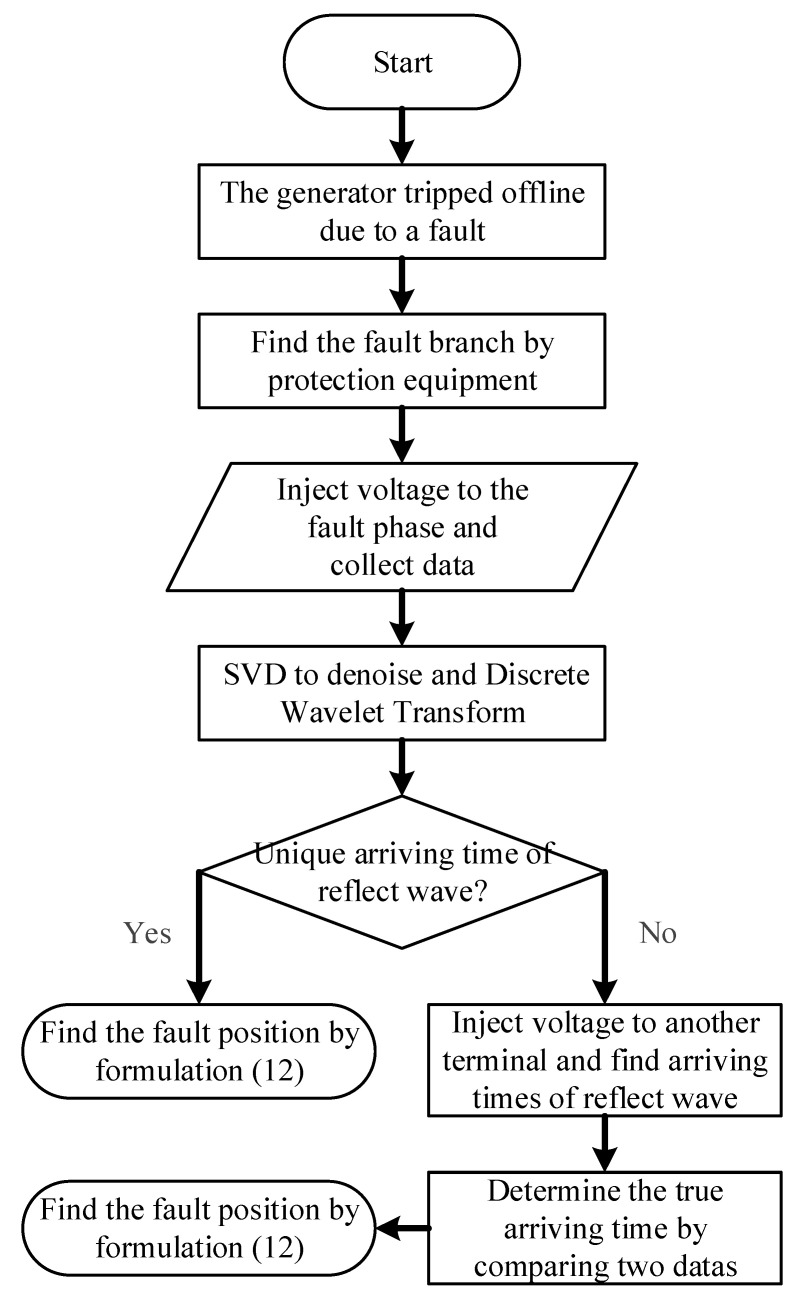
Flowchart of the fault localization process.

The procedure of the localization method for single-phase grounding faults with high resistance in generator stator windings is shown in Figure 7. The fault location methods for lower and higher resistance are shown, respectively.

(1) After determining the fault branch by the protection equipment, inject a segment of DC voltage signal from either side of the stator winding, and simultaneously begin collecting the traveling wave current signal at the injection terminal and the traveling wave voltage signal at the non-injection terminal.
(2) Perform singular value decomposition denoising on the collected voltage and current signals. The key to signal denoising lies in setting an appropriate threshold to ensure that the traveling wave information is preserved during denoising.
(3) Perform discrete wavelet transform on the denoised voltage and current signals, select an appropriate WTMM threshold, and extract the instant when the traveling waves arrive. If a unique traveling wave arrival time can be extracted, use Equation (12) to locate the fault. If multiple arrival times are identified, we inject the signal again at the non-injection terminal and compare the results to determine the arrival time of the traveling wave, completing the fault location process.

In the Baihetan Hydropower Plant, the generator rated voltage is 24 kV, so the 100 V injection adopted in our method is negligible and safe. Maintenance standards also require the test current to not exceed 20 A, and our simulations confirm that this condition is met. Moreover, the signal injection and the subsequent wave reflection/refraction process are completed instantaneously, so the transient stress imposed on the stator winding is minimal.

In terms of practical application, our dual-terminal fault localization scheme can effectively overcome the reduction in accuracy caused by high grounding transition resistance, thus improving the reliability and precision of single-phase stator winding fault location. Compared with online methods such as the third-harmonic-based approach in [11], which indeed provides rapid detection but still requires generator shutdown for inspection and repair, the proposed offline scheme avoids secondary damage and ensures operational safety. Furthermore, unlike some offline methods such as the high-current or resistance-based techniques reported in [14], which may involve complex or even destructive procedures, our method achieves accurate localization through low-level, non-destructive injection. Taken together, the workflow offers a safe, practical, and effective solution to the long-standing engineering challenge of fault location in large generators.

## 4. Simulation and Verification

### 4.1. Simulation Model

The reflected wave from the fault point arrives at the injection terminal earlier than the reflected wave from the non-injection terminal. The velocity of traveling wave is similar to the light velocity, and the length of stator winding is 360 m, thus, after the injection, the reflected wave from the non-injection terminal arrives at the injection terminal in approximately 2.4 μs. To capture the former reflected wave, the time window should be larger than 2.4 μs, and we take it as 5 μs to ensure a time margin.

### 4.2. Fault Location for the High Grounding Transition Resistance

**Figure 8:**
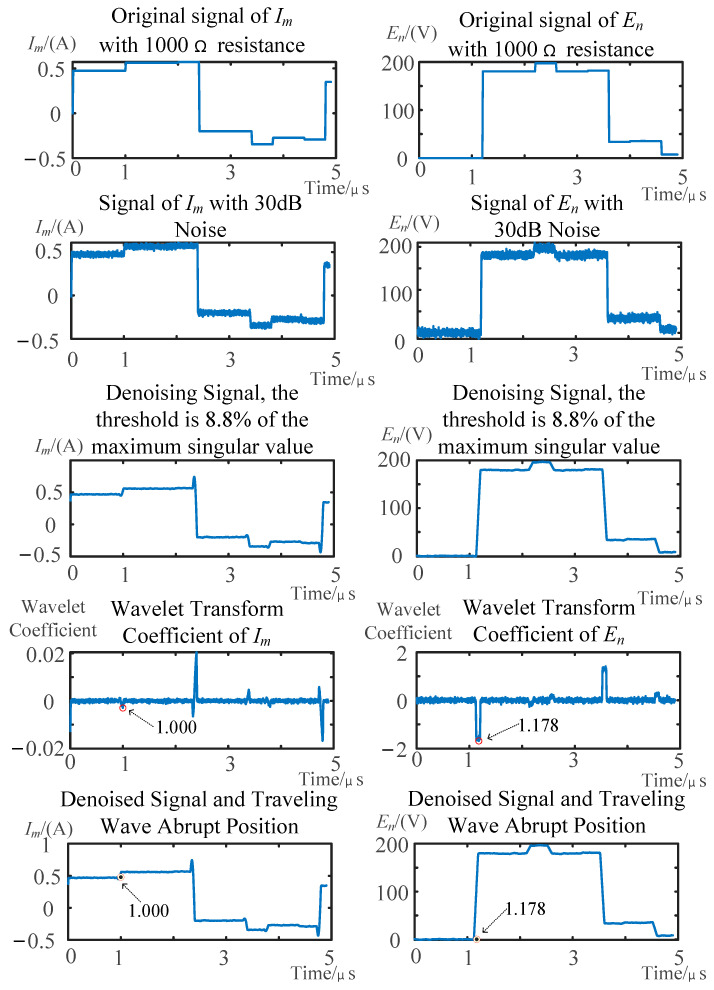
Fault detection of a 1000 Ω transition resistance under noise interference.

**Figure 9:**
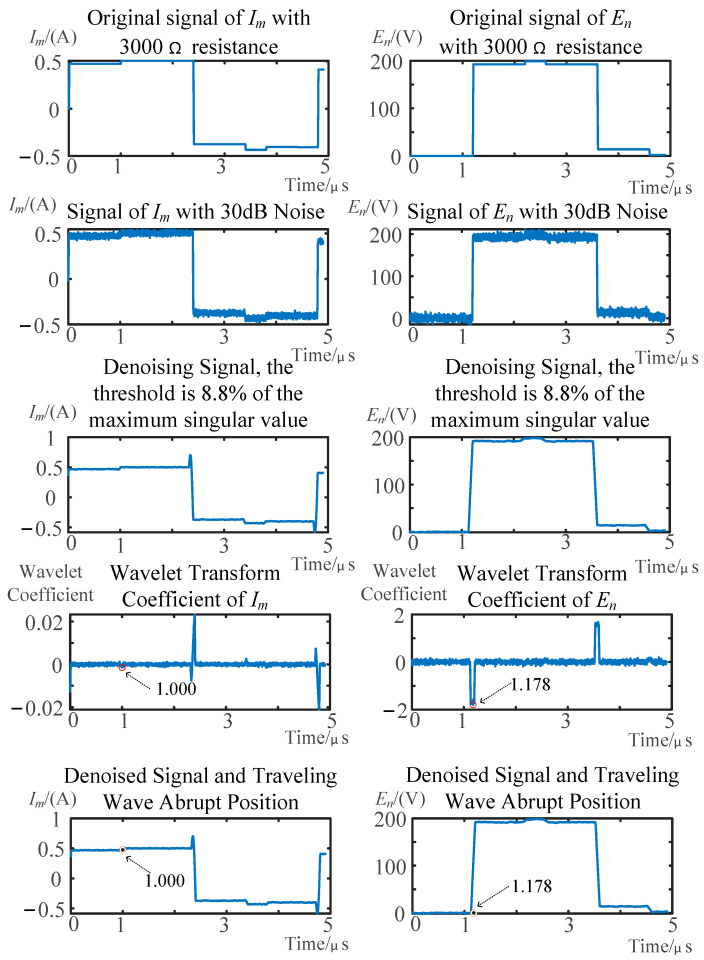
Fault detection of a 3000 Ω transition resistance under noise interference.

The fault localization scheme was validated for stator winding ground faults with transient resistances of 1000 Ω, 1500 Ω, 2000 Ω, 2500 Ω, and 3000 Ω. Figure 8 and Figure 9, respectively, display the current signal at the injection terminal (*I_m_*) and the voltage signal at the non-injection terminal (*E_n_*) under fault resistances of 1000 Ω and 3000 Ω, with the *x*-axis corresponding to the number of sampling points and the *y*-axis representing the voltage and current signal amplitudes. The figures also show the signals with noise, the denoised signals, the wavelet transform results, and the marked arrival times of the traveling waves on the original signal. Table 1 summarizes the calculation results of fault distance under different fault resistances, and the reflected wave WTMM refers to the value of WTMM of injection terminal, caused by the arrival of reflected wave from the fault point.

**Figure 10:**
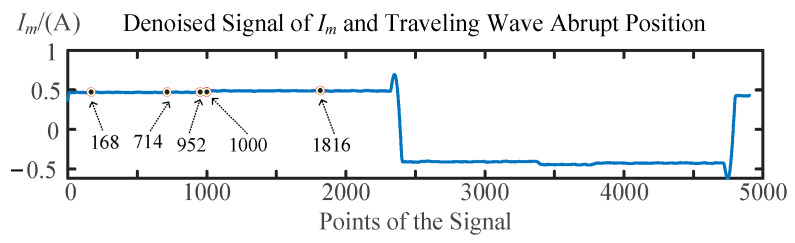
Waveform reflection process when the signal is injected from terminal M.

**Figure 11:**
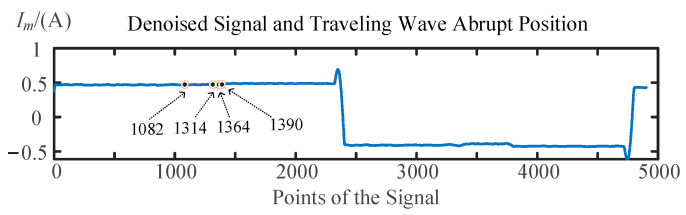
Waveform reflection process when the signal is injected from terminal N.

As the fault resistance continues to increase, for example, at 5000 Ω, the signal mutation caused by the traveling wave’s arrival is already buried within the denoised fluctuations. As shown in Figure 10, injecting a signal from terminal M and extracting the reflected wave signal, multiple suspected arrival times of the traveling waves are obtained through denoising and wavelet transformation, corresponding to fault locations of 25.671 m, 109.1 m, 145.467 m, 152.801 m, and 277.487 m. Similarly, as shown in Figure 11, injecting a signal from terminal N and extracting the reflected signal yields multiple suspected arrival times, corresponding to fault locations of 196.884 m, 161.909 m, 154.372 m, and 150.452 m. The fault distance of 152.801 m measured from terminal M was closest to the fault distance of 150.452 m measured from terminal N, and their average value of 151.6265 m was determined to be the fault location by this method.

Based on this method, the positioning results at different fault locations were calculated separately under transition resistances of 1000 Ω, 2000 Ω, 3000 Ω, 4000 Ω, and 5000 Ω. Here is the calculation formula for the error:

(17) $$Error=\frac{Fault\;Position-Fault\;Location}{Length\;of\;the\;Stator\;Winding}\times 100\%$$

As shown in the Table 2, Table 3, Table 4, Table 5 and Table 6, the fault location method proposed in this paper aimed to identify the fault reflection wave at the fault point for ground faults with high resistance in generator stator windings and white noise caused during data acquisition, by using signal injection and SVD denoising, respectively. The simulation results demonstrated that this location method achieved high positioning accuracy under different fault locations and high-resistance faults. The fault location could help identify the faulty conductor bar of the generator.

### 4.3. Comparison with Alternatives Methods

There are different location methods for generator stator faults, and several typical methods are discussed. For the methods in [9,11], the fault is located after the stator fault occurs and before the generator shutdown, that is, online fault location. These online location methods are rather quick and achieve high accuracy under low-resistance faults; however, they require exact parameters of generator stator, such as equivalent capacitor of windings, which may vary during long-term operations and thus bring location errors.

Because the generator shutdown is unavoidable after the fault occurs, the offline fault location is also important. The binary search method, which is the most common method, works as follows: it divides the fault branch into two equal parts, then use a megohmmeter to measure the insulation resistance of each part; the part with a lower resistance is taken as the new fault branch, and the above operations are repeated until the fault is exactly located. The binary search method does not require complex location equipment and is rather simple and reliable, which is rather suitable for small-scale generators. However, for the large-scale generator, the binary search method requires multiple disassembly operations on the winding, which is rather time-consuming and may bring harm to the winding; thus, novel location methods are required. The offline method in [14] locates the fault using the measured currents, but it has a fault resistance limitation of 1.25 kΩ. The method in [14] and the proposed method both have the characteristics of high location accuracy, no requirement for obtaining winding parameters, and are not harmful to the winding. Compared with the method in [14], the proposed method requires a higher sampling rate and higher precision sensors, which increases the device cost; nevertheless, the proposed method improves the tolerance ability of fault resistance to 5 kΩ, indicating wider application situations.

## 5. Conclusions

High-resistance grounding faults in generator stator windings are notoriously difficult to detect due to their weak fault characteristics. To overcome this challenge, this paper introduces a novel fault location method that combines signal injection with detailed traveling wave analysis. The main conclusions are as follows.

(1) By injecting the DC voltage from one terminal of the fault branch and maintaining the other terminal open-circuited, the voltage at the non-injection terminal and the current at the injection terminal can be used for the subsequent fault location.
(2) The SVD method is used to denoise the acquired voltage and current signals, which ensures the feasibility of the proposed method in practical engineering.
(3) The WTMM can be used to identify the arrival time of traveling wave, and for the fault resistance no more than 3000 Ω, the fault location can be directly calculated using the arrival time of traveling wave of both terminals.
(4) For the high-resistance faults, both terminals are successively injected with DC voltage. The accurate fault location is achieved by comparing the calculation results obtained from the two injection events. The fault location error is no more than 1.02% even when the fault resistance and noise are 5 kΩ and 30 dB, respectively.
(5) The proposed method has characteristics of high fault location accuracy, excellent tolerance to high-resistance faults, no requirement for obtaining winding parameters, and causes no harm to the winding, which are crucial for the fault location of generator stator.

## Figures and Tables

**Table 1:** Fault localization results for different transition resistances.

Grounding Transition Resistance/Ω	Wavelet Threshold	Fault Location/m	Error/%
1000	0.0026	152.801	0.78%
1500	0.0017	151.980	0.55%
2000	0.0018	151.322	0.37%
2500	0.0016	152.801	0.78%
3000	0.0014	152.801	0.78%

**Table 2:** Fault localization results for a 1000 Ω grounding transition resistance.

Fault Position/m	Fault Location/m	Error/%
30	28.52	0.41%
70	70.22	−0.06%
110	110.94	−0.26%
170	172.68	−0.74%
250	253.04	−0.84%
310	310.91	−0.25%

**Table 3:** Fault localization results for a 2000 Ω grounding transition resistance.

Fault Position/m	Fault Location/m	Error/%
30	28.60	0.39%
70	71.38	−0.38%
110	110.82	−0.23%
170	168.08	0.53%
250	248.03	0.55%
310	313.63	−1.01%

**Table 4:** Fault localization results for a 3000 Ω grounding transition resistance.

Fault Position/m	Fault Location/m	Error/%
30	29.60	0.11%
70	69.55	0.13%
110	108.42	0.44%
170	168.94	0.29%
250	248.29	0.48%
310	308.79	0.34%

**Table 5:** Fault localization results for a 4000 Ω grounding transition resistance.

Fault Position/m	Fault Location/m	Error/%
30	30.45	−0.13%
70	68.06	0.54%
110	109.37	0.17%
170	168.34	0.46%
250	248.70	0.36%
310	308.57	0.40%

**Table 6:** Fault localization results for a 5000 Ω grounding transition resistance.

Fault Position/m	Fault Location/m	Error/%
30	29.60	0.11%
70	68.54	0.41%
110	109.25	0.21%
170	170.43	−0.12%
250	249.73	0.08%
310	309.68	0.09%

## Data Availability

The data that support the findings of this study are available from the corresponding author upon reasonable request. However, the availability of some data is restricted as they were used under license from China Yangtze Power Co., Ltd. for the current project and are not publicly available.
